# *MalHaploFreq*: A computer programme for estimating malaria haplotype frequencies from blood samples

**DOI:** 10.1186/1475-2875-7-130

**Published:** 2008-07-15

**Authors:** Ian M Hastings, Thomas A Smith

**Affiliations:** 1 Liverpool School of Tropical Medicine, Pembroke Place, Liverpool, L3 5QA, UK; 2 Swiss Tropical Institute, Socinstrasse 57, CH-4002 Basel, Switzerland

## Abstract

**Background:**

Molecular markers, particularly those associated with drug resistance, are important surveillance tools that can inform policy choice. People infected with *falciparum *malaria often contain several genetically-distinct clones of the parasite; genotyping the patients' blood reveals whether or not the marker is present (i.e. its prevalence), but does not reveal its frequency. For example a person with four malaria clones may contain both mutant and wildtype forms of a marker but it is not possible to distinguish the relative frequencies of the mutant and wildtypes i.e. 1:3, 2:2 or 3:1.

**Methods:**

An appropriate method for obtaining frequencies from prevalence data is by Maximum Likelihood analysis. A computer programme has been developed that allows the frequency of markers, and haplotypes defined by up to three codons, to be estimated from blood phenotype data.

**Results:**

The programme has been fully documented [see Additional File 1] and provided with a user-friendly interface suitable for large scale analyses. It returns accurate frequencies and 95% confidence intervals from simulated dataset sets and has been extensively tested on field data sets.

**Conclusion:**

The programme is included [see Additional File 2] and/or may be freely downloaded from [1]. It can then be used to extract molecular marker and haplotype frequencies from their prevalence in human blood samples. This should enhance the use of frequency data to inform antimalarial drug policy choice.

## Background

The identification of molecular markers (mutations) associated with drug resistance in *P. falciparum*, and the ability to detect these markers in the blood of infected people, means that large-scale population surveys can be used to infer the likely efficacy of antimalarial drug treatment regimes [2]. This allows their use in large scale surveillance surveys [3]. These surveys measure, and generally report, the prevalence of the marker i.e. the proportion of patient blood samples in which the marker is detected. This is clinically-useful information (it is related to a patient's probability of failing drug treatment) but it is less appropriate as a public health surveillance tool. It is the frequency of the drug resistant mutation, defined as the proportion of parasite clones in which the marker is present, and the rate at which it is increasing which determines the likely time before a drug becomes ineffective and requires replacement. Prevalence and frequency may differ markedly because several parasites clones often simultaneously co-infect the same patient: for example if patients have three clones (a 'multiplicity of infection' (MOI) of three) the frequency of the mutation among the parasites may be 10% but among patients its prevalence will be almost three times higher ≈ 3 × 10% = 30% because each clone has a chance of bearing the marker (the true value, assuming statistical independence of clones, is actually 1-(1-0.1)^3 = 0.27 = 27%).

**Figure 1:**
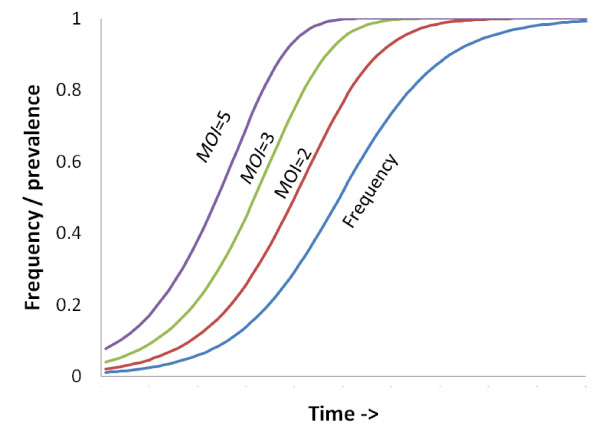
**How prevalence depends on frequency and multiplicity of infection (MOI)**. The marker has a 10% selective advantage over the wildtype form and its frequency is increasing over time. The corresponding prevalence of the marker is shown over the same time scale assuming each human in the population contains either 2, 3, or 5 malaria clones (MOI = 2,3,5).

This has several consequences: (a) Prevalences depend on MOI so, unlike frequency, they are not directly comparable across regions with different epidemiology (Figure 1); (b) Prevalences have different dynamics compared to frequency: they increase rapidly in the early spread of resistance but less slowly at later times (Figure 1); (c) The selection coefficient driving resistance is a key population genetic measurement but can only be estimated from a time series of frequency data [4].

A further drawback of using prevalences is that they do not measure the frequencies with which combinations of alleles, (haplotypes) occur together in the same parasite. It is these haplotype frequencies that most directly measure the probability that a parasite is genetically resistant. For example, mutations in codons 51, 59 and 108 in *dhfr *all affect a parasite's ability to survive treatment with antifolate drugs (reviewed in [5]), and the probability that parasites will survive in a mixed infection depends on whether these mutations are present in the same genome or not. The prevalences of mutations at each codon cannot be directly translated into haplotype frequencies. For example, a patient may be infected by two malaria clones and genotyping reveals the presence of only mutants at position 108 and the presence of both mutant and wildtype at positions 51 and 59; consequently, it is impossible from these data to distinguish whether the clones are (i) mutant at 108 in one clonal haplotype and mutant at 108+51+59 in the other haplotype, or (ii) mutant at 108+51 in one clone haplotype and mutant at 108+59 in the other haplotype. The situation becomes even more complex as MOI increases.

Statistical approaches for estimating gene and haplotype frequencies in the presence of uncertainty use Maximum Likelihood methodology and are described in standard population genetic textbooks such as Hartl & Clark [6]) and in journal reviews such as Williams & Dye [7]). This approach was used by Hill and Babiker [8] to estimate MOI in blood samples from Tanzania. If the MOI is known, then estimation of allele frequencies for resistance markers in malaria is analogous to that of estimating frequencies from phenotype data in a polyploid organism, where the MOI is equivalent to the ploidy. Such estimates have been reported in a study of chloroquine resistance markers in Tanzania [9] and software for carrying out such estimation is available at [10].

The problem of estimating haplotype frequencies from such ambiguous genetic data is somewhat more complicated. An appropriate method is, once again, to use ML methodology. In essence the ML approach is to initially guess the frequency of haplotypes, measure how consistent these frequencies are with the observed prevalence of combinations of mutations in the blood samples, and continue changing and improving estimated values of haplotype frequencies until they provide the best match to the data. This paper describes a freely-available computer programme that uses these techniques to estimate both allele and/or haplotype frequencies from prevalence data. It has two main advantages over existing methodologies.

Firstly, it can simultaneously analyse all the samples in a dataset irrespective of their levels of MOI/ploidy. Haplotypes can be directly observed in samples with only a single infection i.e. where MOI is 1. Samples with MOI of 2 are diploid (the sample contains two haplotypes) and haplotype frequencies could be inferred in this subset of the data using existing software (e.g. [11]) designed to analyse samples from diploid organisms such as humans, mice, drosophila (where, obviously, one haplotype comes from the mother and one from the father). To our knowledge no software exists to infer haplotype frequencies where ploidy (MOI) exceeds 2 and our software is explicitly designed to allow this. Our software therefore allows simultaneous analysis of the entire dataset including samples where MOI exceeds 2.

Secondly, existing methods for estimating frequency at a single SNP in malaria samples (e.g. [8,9] cited above) assume perfect information of the constituent haplotypes. For example, if a sample contains four haplotypes of type 'A' and 1 of type 'B' then these analyses assume the sample would be correctly genotyped as 'AB'. In practice 'minor genotypes' are often not detected and the sample would be misclassified as type 'A'. This non-detection may be due to the minor clone signal being swamped during PCR amplification and/or because many genotyping protocols state that 'minor signals' (e.g. "genotypes returning a signal less than 20% of the largest peak") be ignored; both situations result in a 'mixed' genotype infection being erroneously recorded as single genotype. Our programme allows this effect to be incorporated into the analysis and again, to the best of knowledge, is unique in this respect.

The programme is designed to be flexible and could be used to analyse similar genetic datasets obtained from other organisms whose samples vary in ploidy. Analysis of samples containing multiple genotypes of other infectious agents is the obvious example, but whole organisms which vary in ploidy level could also be analysed using our approach. However the software was specifically designed to analyse malaria in blood samples and herein will only be discussed within this context.

## Methods

**Figure 2:**
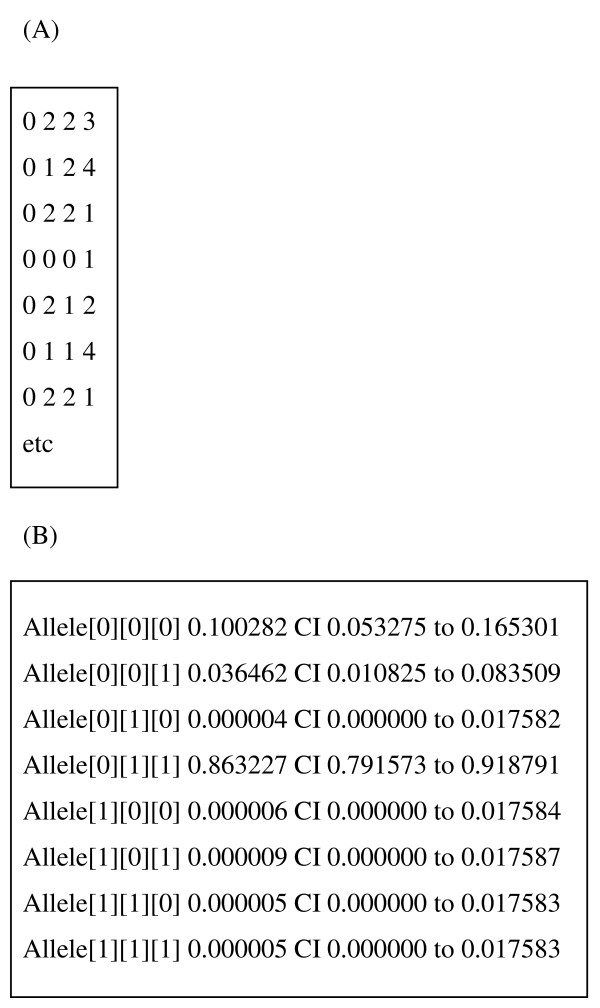
**Input and output formats for MalHaploFreq**. (A) Input file format. Each line corresponds to a single blood sample. The first three indices are phenotypes at up to three codons where "0" indicates only wildtype is present, "2" mean only mutant are present,"1" means both wildtype and mutations are present. The fourth index is the multiplicity of infection (MOI). Assuming, for example, that *dhfr *is being analysed and that codons 1,2 and 3 represent positions 51, 59 and 108 respectively, then the first sample has only wildtype at position 51, only mutants at positions 59 and 108, and its MOI = 3. The second sample has only wildtype at position 51, has both wildtype and mutant and position 59, only mutant at position 108, and its MOI = 4. And so on throughout the input dataset. Missing data are indicated as '99' in the indices. (B) Output format. The indices represent codon genotypes, the first corresponding to codon 1, the second to codon 2 and the third to codon 3; within these brackets, "0" indicates wiltype and "1" indicates mutant. Assuming, as above, that *dhfr *is being analysed and that codons 1,2 and 3 represent positions 51, 59 and 108 respectively, then a haplotype mutant at only position 108 is encoded [0][0][1] and its estimated frequency is 3% with 95% CI of 1% to 8%. The 'double' mutant haplotype with mutations at positions 59 and 108 is encoded [0][1][1] and its estimated frequency is 86% with 95% CI of 79% to 92%. And so on.

The programme was written in C for ease of portability across PC, Macintosh and UNIX computer operating systems. A comprehensive users' manual accompanies the programme [see Additional File 1] and gives greater details of the underlying methodology as well as details on how to run the programme, interpret the data, and more general advice on decisions that have to be made is setting up the analysis. The programme (PC version) and manual can be freely downloaded from [1] and the basic format of input and output is shown on Figure 2.

The programme will estimate haplotype frequencies defined by mutations at up to three codons. This limit was dictated by the fact that the complexity of the calculations rises exponentially with the number of codons. For example, analysis of haplotypes defined at 3 codons has to consider 3^3 ^= 27 blood-sample phenotypes and estimate 2^3 ^= 8 haplotype frequencies, while extending the analysis to haplotypes defined at 5 codons requires considering 3^5 ^= 243 blood-sample phenotypes and estimating 2^5 ^= 32 haplotype frequencies. A further reason for limiting haplotype definitions to a maximum of three codons is because it is rarely necessary in practice to analyse more than three codons simultaneously. For example, mutations in *dhfr *invariably accumulate in sequence, first at codon 108, then 51 or 59 then at 164 so if analysing the frequency of haplotypes containing the 164 mutation, it can be safely assumed that all haplotypes containing the 164 mutation are also mutant at codon 108 (and probably also mutant at positions 51 at 59), so codon 108 can be omitted from the analysis. Note also that if a haplotype of interest is defined at separate, unlinked genes such as *dhfr *+ *dhps *(e.g. the *dhfr*108+51+59 with *dhps*437+540 'quintuple' mutant haplotype) or *crt76*+*mdr86 *haplotype, it is unnecessary to estimate the frequency of haplotypes defined by both loci: it is invariably sufficient to estimate the haplotypes at each locus separately and then assume linkage equilibrium between the loci (this is discussed in more detail in the programme user notes).

### Algorithm

The programme is initialised internally by generating random haplotype frequency estimates and employs a 'hill climbing' routine to improve these estimates until it arrives at the ML estimate of haplotype frequencies. The estimated frequency of each haplotype is then systematically varied from its ML estimate and its 95% confidence interval (technically, a 95% support interval) is defined by the points at which the Log likelihood falls 2 units below the ML value [8].

The Log Likelihood appropriate for any given combination of parameter estimates is obtained as follows, using the haplotype and phenotype coding described in Figure 2. The programme considers each possible MOI in turn and cycles through all the combinations of the eight haplotypes that can occur within that MOI. For example, if MOI = 5, one possible combination would be 3 haplotypes of type [1][1][1] (i.e. mutant at all codons), 1 haplotypes of type [0][1][1] (i.e. wildtype at codon 1, mutant at codons 2 and 3) and 1 haplotypes of type [1][1][0] (i.e. mutant at codons 1 and 2, wildtype at codon 1). This would result in a blood sample phenotypes of [1][2][1][5] i.e. wildtype and mutants present at codon 1, only mutant at codon 2, wildtype and mutants present at codon 3, with MOI of 5. The probability of getting this combination is obtained from the multinomial distribution i.e.

(1) $$\left(\begin{array}{l} 5\\ 3,1,1 \end{array}\right)3^{x}1^{y}1^{z}$$

where *x*, *y*, *z *are the current estimates for the frequencies of haplotypes [1][1][1], [0][1][1] and [1][1][0] respectively and the multinomial coefficient is

$$\left(\begin{array}{l} 5\\ 3,1,1 \end{array}\right)=\frac{5!}{3!\;1!\;1!}$$

A running total of probabilities of observing the various blood phenotypes is kept stored in an array prob [*p*] [*q*] [r] [*s*] where the final index, *s*, is MOI. So in the above example the running total kept in prob[1][2][1][5] would be incremented by the solution of Equation 1.

This is a very flexible approach because it examines each possible combination of clone haplotypes and works out the resulting phenotype that would be observed in the dataset rather than the true phenotype. So if, for example, minority clones are missed during the genotyping this is where the effect is incorporated. In the above example, assume clones present at frequency less than 0.33 in the sample are missed in the genotyping (this value is user-defined) then the 'true' phenotype [1][2][1][5] would actually be observed as [2][2][2][5] because the single clones with wildtype at codons 1 and 3 are below the detection limit of 0.33; consequently the value of prob[2][2][5][5] (rather than [1][2][1][5]) would be incremented by the solution of Equation 1.

### Testing

The programme has been used to estimate one-, two- and three-codon haplotypes on 16 unpublished datasets collected from Papua New Guinea (PNG) and Tanzania; each was subject to 7 separate analyses (spread over 4 loci: *crt*, *mdr*, *dhfr *and *dhps*), making 112 analyses in total. The programme ran smoothly, correctly identified inconsistencies in the data (e.g. identified samples encoded as having mixed mutant/wildtype infections in a single-clone infection) and gave sensible output. More specific and stringent testing was done in three parts.

A routine was built into the programme to simulate a dataset of the same type and size being analysed. The routine records exactly how many haplotypes of each type enter into the simulated dataset, ensuring the 'true' haplotype frequencies are known in the simulated dataset. It then invokes the main programme to see how well it estimates these 'true' simulated frequencies and whether the 'true' values fall within the 95% confidence interval. The user can command the programme to do this numerous times (e.g. 1,000) to check the programme accuracy: it prints out 'true' frequency, estimated frequency with 95% confidence intervals (CI) and whether the 'true' frequency falls within the 95% CI. At the end of the process it prints out how often the 'true' frequencies fall within the 95% CI. Obviously if the programme is working well this should happen about 95% of the time, and this was observed to be the case.

Single codon haplotype frequencies were available for 32 of the PNG/Tanzania analyses (the 16 datasets each analysed at *crt*76 and *mdr*86). These were obtained using a different algorithm and software (a Bayesian approach using WinBugs [9]). Both our analyses gave the same results. Extending the analysis to 2- and 3-codon haplotypes in MalHaploFreq were consistent with the single codon results in two important respects. Firstly, if there was no genetic variation at the additional codons then only two haplotypes were identified with frequencies identical to the one-locus results (although CI were slight larger). Secondly, if genetic variation was present at the additional codons then summing estimated haplotypes into two classes, corresponding to the single-codon haplotypes, gave identical frequencies.

The programme has an option to run the analyses numerous times from different starting frequencies. The starting frequencies used to initialise the program are generated at random and updated and improved to converge on final estimates. There is no guarantee that the programme will always converge on the same set of estimated frequencies, so it is important that the user can test that this is the case.

## Results

Extensive analyses of simulated datasets revealed that the 95% confidence limits were correctly estimated i.e. that the 'true' frequencies in the simulated datasets fell within the 95% CI in more than 95% of analyses. In fact around 98% to 99% of true frequencies fell within the 95% CI, presumably because the approximation used by maximum likelihood to calculate confidence intervals (i.e. a drop in 2 log likelihood units) is conservative. The 95% CI therefore err on the side of caution by being slightly too wide.

The 112 PNG and Tanzanian analyses were each re-analysed from 1000 initial haplotype frequency estimates to check that all 1000 re-analyses converged on the same estimates of haplotype frequency. There was one instance (in a three-codon analysis) where there appeared to be two points of convergence, the analysis converging on a single peak of lower LL in approximate 10% of the runs while the remainder converged on a peak of higher LL in the remaining 90% of cases. The differences in estimated frequencies were large, one estimated haplotype frequency increasing from 0.37 to 0.5 with a corresponding fall from 0.47 to 0.33 in the other main haplotype. Such 'false peaks' are rare in ML analyses but do occur. There is an option built into MalHaploFreq to check convergence from a user-defined number of different starting frequencies and users are strongly recommended to perform this check.

## Discussion

**Figure 3:**
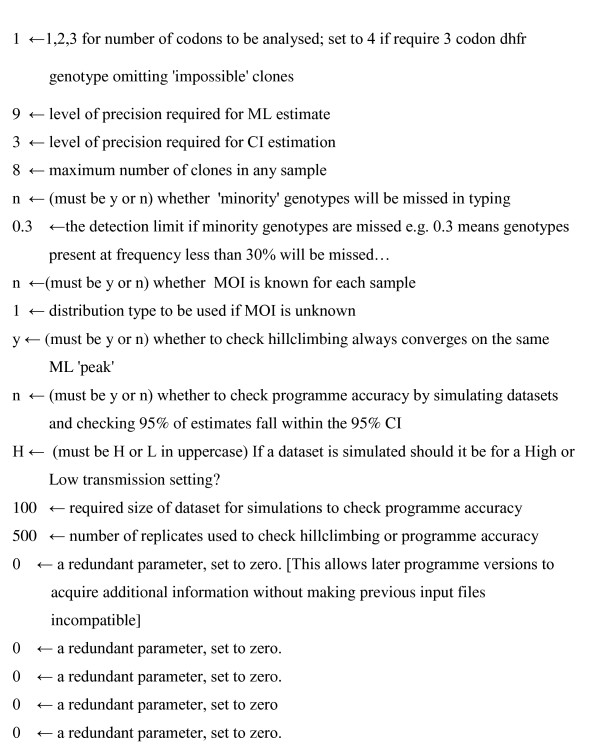
**Example parameter file for MalHaploFreq**. Parameters required to run MalHaploFreq are read from an external file 'MHFparameters.txt' in the following format. Descriptions for the parameter values are provided to the right of the arrow and are self-explanatory. The parameter values are to the left of the arrow; for example, the first parameter instructs MalHaploFreq to investigate haplotypes defined at only a single codon.

The programme appears to perform well in estimating haplotype frequencies from both field and simulated datasets. It reads input parameters from a separate file rather than from a graphical interface and an example of an input file is provided on Figure 3. The advantage of this strategy is that analyses can be automated into batch files. For example part of a DOS batch file may read as follows:

del MHFdatafile.txt

del MHFparameters.txt

copy VillageAdatafile.txt MHFdatafile.txt

copy VillageAparameters.txt MHFparameters.txt

MalHaploFreq.exe > VillageAoutput.txt

The first two lines delete the default data and input parameter files. The next two lines copy the data for Village A into the default MalHaploFreq datafile (see Figure 2) and copy the required parameters for analysing Village A into the default parameter file (Figure 3). The final line runs MalHaploFreq.exe and dumps the screen output (using the '>' command) into an appropriately-named output file for future reference. A typical project will require many separate ML analyses, for example, many villages analysed at several loci with 1, 2, or 3 codons being analysed. Each analysis can be included in an automated analysis simply by setting up the data and parameter files required for each analysis and copying, pasting and editing this blocks of 5 lines into a larger DOS batch file.

This automation has two large advantages. Firstly, the analyses can be initially run rapidly with low levels of precision primarily to check the analyses proceed correctly and that any inconsistencies in the data identified by the program can be corrected. Contemporary methodology (such as genotyping chips) often produces large amounts of data on different loci and inconsistencies identified in one analysis can alter results obtained in the other; a common experience was to find that MOI was originally encoded as 1, indicating that a single clone was present, but later analysis of other loci revealed some codons to be mixed wildtype+mutant indicating that at least 2 clones must be present. The datasets could then be checked and revised if required (so that MOI > 1) and the automated analysis easily repeated. The second advantage comes after the datasets are cleaned and ready for the definitive analyses. High levels of precision in frequency estimates and CI require considerable computer time, and there are associated computer-intensive checks to be made, particularly checking that the programme convergences on the same solution from numerous starting parameter values (1000 is recommended). The most convenient way of running these analyses is to download them onto a spare computer (typically a laptop) and leave an automated analysis ruining over the required period (for example, it required three weeks to perform and check the 112 PNG and Tanzanian analyses).

## Conclusion

The use of molecular markers to track the spread of drug resistance and to guide policy requires that the frequency of mutations and haplotypes be calculated rather than their prevalence. An appropriate method to estimate their frequencies from blood sample data is by Maximum Likelihood techniques. This paper describes a flexible, freely downloadable computer program which implements this approach.

## Authors' contributions

IH designed the algorithm and wrote the programme. IH and TS tested it against field data and co-wrote the manuscript.

## Supplementary Material

Additional File 1 User manual for MalHaploFreq. Click here for file

Additional File 2 executable programme compiled for use on DOS or windows Click here for file
