# Supplementary material for: MalHaploFreq: A computer programme for estimating malaria haplotype frequencies from blood samples
Source: Malar J. 2008 Jul 15;7:130. doi: 10.1186/1475-2875-7-130 (PMC2490701; doi:10.1186/1475-2875-7-130)
Supplement: Additional File 1 — User manual for MalHaploFreq. [file 1475-2875-7-130-S1.pdf]

MalHaploFreq

A computer program for estimating malaria allele and haplotype <u>frequences</u> from their <u>prevalences</u> in blood samples using Maximum Likelihood methodology

Written/collated by Ian Hastings, Liverpool School of Tropical Medicine.

Notes Version 1.1 for
Programme Version 1.1.1

Contents:

1. Basic principles: using Maximum Likelihood (ML) methodology to estimate allele and haplotype frequencies from blood samples (page 3).

2. Decisions required before data analysis (page 7).

3. *Running the analysis (page 10).*

4. *Checking the analysis: checks you should make (page 16).*

5. Checking the analysis: checks I have made (page 18).

6. How to tell if frequencies differ between datasets (page 19).

7. Acknowledgements (page 21).

Appendix 1: The MalHaploFreq algorithm (page 22).

Appendix 2. Alternative algorithm (page 26).

Appendix 3. The original version of the Smith and Penny algorithm (page 29).

Appendix 4. Simple tests to check programme is working (page 31).

Appendix 5. History of programme versions and updates (page 32)

References (page 33).

1. Basic principles: using Maximum Likelihood (ML) methodology to estimate allele and haplotype frequencies from blood samples.

In natural systems the same observation can arise in several different ways, making the data ambiguous and 'standard' statistical analysis problematic. This often occurs in genetic datasets:

- A classic case in population genetics is the familiar ABO system of blood groups where A and B types can be detected but O simply reflects the absence of A and B. Humans are diploid so people of blood type A and B are genetically ambiguous: people with blood group phenotype A can be genotype AA or AO and people of blood group phenotype B could be genotyp BB or BO [people with groups O and AB are unambiguous and have genotypes OO and AB respectively].

- An analogous situation arises in malaria when examining a single mutation e.g. the *crt*K76T mutation or the *dhfr*108 mutation. For example a person with four malaria clones may contain both mutant and wildtype forms of a marker but it not possible to distinguish the relative frequencies of the clonal genotypes i.e. 1:3, 2:2 or 3:1.

- A more complicated case occurs in haplotypes defined at more than one codon. For example, a patient may be infected by two malaria clones and genotyping reveals the presence of only mutants at position 108 and the presence of both mutant and wildtype at positions 51 and 59; consequently, it is impossible to distinguish whether the clones are (i) mutant at 108 in one clonal haplotype and mutant at 108+51+59 in the other haplotype, or (ii) mutant at 108+51 in one clone haplotype and mutant at 108+59 in the other haplotype. The situation becomes ever more complex as MOI increases.

Put simply, we can measure the <u>prevalence</u> of mutations in *P. falciparum* but, because of the complication of multiple infections and resulting genetic ambiguitiy, we cannot directly estimate their <u>frequency.</u>

The standard way of analysing this type of dataset is by Maximal Likelihood (ML). A general introduction to this methodology can be found in (1, 2) and its application to this problem in malaria is described in (3, 4). Basically an ML analysis consists of initially guessing the values of the required parameters such as haplotype frequencies, measuring how consistent these frequencies are with the observed prevalence of mutations in the blood samples, and then to continue changing and improving estimated values of mutation/haplotype frequencies until they provide the best match to the data. Eventually a combination of parameters values (haplotype frequencies) is obtained that is the <u>most likely</u> combination to have given the observed data set. 95% confidence intervals around these estimates are then obtained by varying the parameter values away from the best estimate.

A simple example is shown on Figure 1 in which the parameter of interest is the frequency of the *P. falciparum dhfr108* mutation. Its frequency is varied along the Xaxis and the corresponding likelihood of observing the dataset is plotted on the Yaxis. The 95% confidence limit occurs when the likelihood is less than 2 log units below the maximum likelihood. So in the example given on Figure 1 the best estimate of *dhfr108* frequency is 0.45 and 95% CI is approx. 0.35 to 0.55.

This illustrates the general principle involved in ML analysis but is the simplest possible case because only a single parameter (*dhfr*108 mutant frequency) is being estimated. If the analysis is extended to haplotypes defined at three codons (e.g. *dhfr* 108, 51 and 59) then there are 3^2=8 different haplotypes and Figure 1 would have to be in 7 dimensions (because only 7 frequencies need to be varied, the eight frequency must be fixed to ensure the frequencies add up to one). It is obviously impossible to show a 7 dimensional graph but the calculations proceed in the same way: all 7 haplotype frequencies are systematically varied to find the set of frequencies with the best match to the observed data set. These are the ML estimated frequencies. The log likelihood (LL) is noted at this point and each of the 8 frequencies are then changed until the LL falls 2 units below the maximum at which point the 95% confidence intervals have been identified.

On a technical note: In practice it is unrealistic to examine all possible combinations of parameters so a ‘hill climbing’ method is used (Figure 1B). Each parameter is varied in turn (increased or decreased in value) to see whether it increases the likelihood. If it does, the new value became the current best estimate, and the next parameter is varied and so on. This method eventually converges on the best combination of parameters.

The process is conceptually simple but requires considerable amounts of computer time, hence the development and distribution of this program.

Figure 1. The maximum likelihood (ML) method. In this simple example each person has only 1 malaria clone is his/her blood. There are 100 samples in the dataset and 45 of them contain the mutant.

(A) How ML calculates best estimates and 95% confidence intervals. ML works by considering each possible frequency of the mutation (along the X-axis) and calculating the logarithm of the likelihood (LL) of obtaining that dataset if the assumed frequency was correct (using the binomial distribution in this case). Not surprising the maximum likelihood occurs when the assumed frequency is 0.45 and this is the ML best estimate. The 95% confidence interval (or 95% support interval) is when the log likelihood is less than 2 log units below the maximum, 0.35 to 0.55 in this case.

(B) Hill climbing methods. The relationship between estimated frequency and LL is often mathematically complex or unknown so is illustrated by a dotted line. Hill climbing works by guessing the initial frequency (0.2 in this case) and changing its value to see if the change increases or decreases LL. If the change increases LL (indicated by green arrows in this example) the altered frequency becomes the current best ML estimate. If the change decreases LL (red arrows) the change is ignored and the previous value is retained. The process is repeated until the Hill climbing algorithm eventually finds the maximum likelihood. 95% CI are found as above by altering each frequency until it falls 2 log units below the maximum LL.

Hill climbing is used by this programme because several (unto eight) haplotype frequencies need to be investigated and there may be several malaria clones in each blood sample. Consequently, it is not possible (or extremely complicated) to work out an algebraic relationship between the haplotype frequencies and LL so a hill climbing algorithm is employed

Figure 1(A).

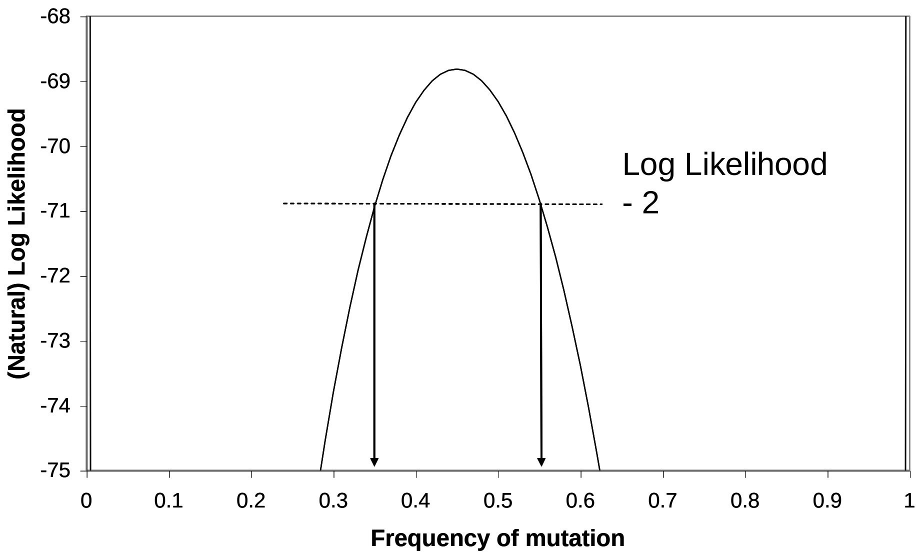

Figure 1(B).

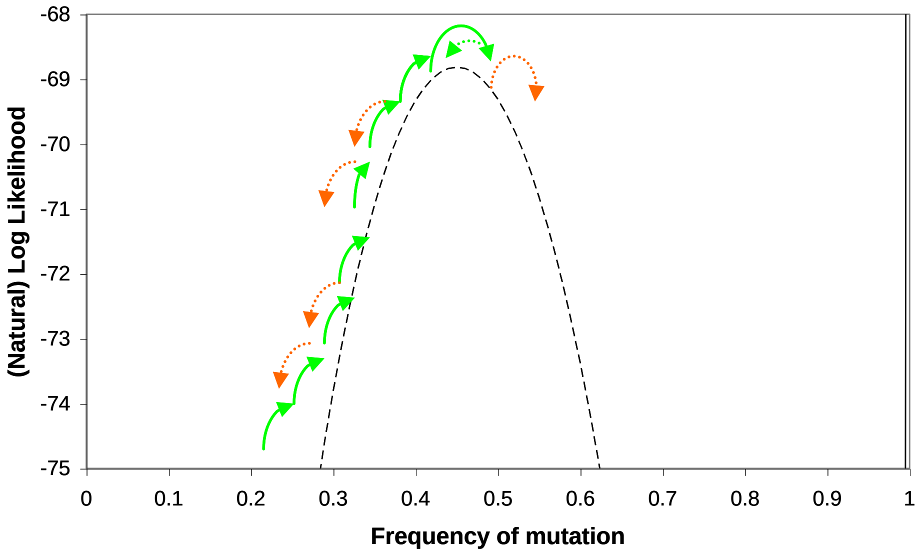

2. Decisions required before data analysis.

(i) How many codons to analyse?

The programme analyses haplotypes defined at 1, 2 or 3 codons.

I am reluctant to expand the programme to analyse more than 3 codons because:

- The complexity increased exponentially, there being 2^n haplotypes for *n* codons. For haplotypes defined at three codons *n*=3 so there are 8 haplotypes (and three of the *dhfr* haplotypes are often ignored as being 'impossible' i.e. 51, 59 and 51+59, meaning only 5 *dhfr* haplotypes are analysed). For haplotypes defined at 5 codons 32 different haplotypes have to be estimated. The confidence intervals will presumably get larger, the programme will take longer to run, and technically the programme will get much more complicated

- It is not clear what benefit, if any, would accrue from increasing the number of codons. For example, if investigating the *dhfr*164 mutation, it is known that it invariably occurs in the presence of mutations at *dhfr* codon 51, 59 and 108 so it is possible to ignore the other codons and simply calculate the frequency of the *dhfr*164 mutation. Similarly for *pfcrt* there are mutations at many sites but most analyses will concentrate on the K76T, for *mdr* it will be codon 86 and so on.

Human nature dictates that if you have genotyped lots of codons it is difficult to simply ignore some of them but unless there are pressing reasons why more than 3 codons have to be simultaneously analysed I am reluctant to further expand the programme.

Similarly, think carefully about how many codons to analyse. Just because you can analyse 3 codons simultaneously does not mean you have to analyse all three. Once the data are collected it is only natural to want to analyse them as much as possible. There is nothing wrong with this strategy as a basic investigation but be careful when publishing them. For example, mutations in *crt* are known to affect CQ resistance. The K76T is thought to be the key mutations but there are several more scattered over other sites. It makes sense to estimate the frequency of the K76T alone: only a single codon is analysed and this should reduce confidence intervals. You could then expand the analysis to look at lots of other haplotypes but ask yourself what this will add to the analysis when publishing because the referees will certainly do so.

(ii) Will the analyses be constrained by sample size?

There is an option to simulate datasets to check the accuracy of the analysis (see later). Simulations have shown that analyses may become erratic at small sample sizes but is robust at larger sample sizes. You should always simulate datasets to check for robust analyses, see later. The obvious question is how big a sample size is sufficient? The same question arises in multiple and logistic regression so, in the absence of any better guidance, I suggest you use the same general rule of thumb i.e. that you need at least 10 observation for each entity (haplotype) that you are trying to measure. So analysis of a single codon, gives 2 haplotypes so minimum sample size is 20 (only

one frequency is actually being estimated, the other is constrained to sum to unity, but its best to be conservative). Analysing two codons gives 4 haplotypes, so minimum sample size is 40. Analysing four codons gives 8 haplotypes, so minimum sample size is 80. Analysing the restricted set of 5 *dhfr* haplotypes means minimum sample size is 50.

(iii) Mutations in different genes.

It is tempting to want to analyse haplotypes defined at codons in more than one gene e.g. the *dhfr*108+*dhfr*51+*dhfr*59+*dhps*437+*dhps*540 'quintuple mutant' or the *crt*76+*crt*220+*mdr*80 ‘triple’ mutation. These are true haplotypes within individual parasites but they consist of mutations in separate, unlinked genes. This makes things much more complicated and involves the concept of linkage disequilibrium (LD). LD measures the non-random association between mutations in different genes (e.g. *dhfr* and *dhps*) that occurs within individual parasites as a balance between two opposing forces

- Drug pressure acts to increase LD because mutations in different genes become (temporarily) associated due to their joint ability to survive drugs.
- Genetic recombination during the obligate sexual phase of the malaria lifecycle acts to randomise the mutations in separate genes thereby destroying LD.

The magnitude of LD therefore depends on local clinical practice (which determines the level and pattern of drug use) and local malaria epidemiology (which determines the frequency of mixed infections and consequently the amount of recombination).

In effect, by looking at haplotypes involving more that one gene you have moved from the realms of molecular genetics to the realm of population genetics. LD and malaria population structure is a hugely controversial area and is best avoided unless you have the requisite specialist technical knowledge (and possibly best avoided even then).

The best way to analyse such data is probably to estimate the haplotype frequencies at each locus separately and then predict the joint haplotype frequency assuming they are in linkage <u>equilibrium</u> (i.e. randomly associated) with a suitable caveat e.g.

“The estimated frequency of *dhfr*108+51+59 haplotype was 67% and that of the *dhps*437+540 haplotype was 50%. This leads to estimated frequency of the quintuple mutation of 0.67x0.5 = 32%. Note that this is likely to be a slight underestimate of the true quintuple frequency because non-random association of the mutations (linkage disequilibrium) may have arisen as a consequence of drug pressure and local malaria epidemiology”.

This should satisfy reviewers that you are aware of the problem and most specialists would appreciate why you have taken a simple approach.

If you have a large number of single-clone infections you could estimate LD directly and bring it into your calculations but you need some population genetic knowledge to do this.

The above is only a suggestion and there may well be different and better approaches to this problem of LD; I'm happy to hear of alternatives.

Note that in principle you could estimate LD using the programme e.g. by estimating frequency of *mdr*80 alone, *crt*76 alone, and joint *mdr*80+*crt*76 haplotype frequency but I don't even want to think about how you could attach standard errors or confidence intervals to the estimate (they are usually constructed by permutation test using specialist computer software such as Popgene or Arlequin).

(iv) <u>*Calculating dhfr haplotype frequencies*</u>

Haplotypes at this locus are usually defined by mutations at codons 108, 51 and 59 which makes 8 haplotypes in total. However experience has show that certain haplotypes are so rare that their frequencies are negligible and we can ignore them:
Possible: wildtype, 108, 108+51, 108+59, 108+51+59
Negligible: 51, 59, 51+59
Analysing 5 haplotypes rather than 8 cuts run time and should hopefully reduce confidence interval.

There is a user-defined option to analyse all 8 haplotypes or just the five 'valid' ones.

(v) Precision of the analysis.
There are user-defined inputs that instruct the programme to estimate the frequencies and confidence intervals to a given level of precision (number of decimal points). There is a nasty pitfall here for the unwary (I know because I fell into it): it is necessary to get a very good estimate of frequencies not to obtain estimates to 10 decimal points, but because it is essential that the program precisely finds the maximum LL value i.e. finds the very top of the 'hill' of likelihood (Figure 1b). If this ML is not precisely measured then its value will be too low and the confidence interval (defined as this ML minus 2) will be too wide. I therefore suggest you employ the following levels of precision according to the type of analysis:

- Initial analysis to check the data are processed correctly and no data errors are found: set both ML and CI levels of precision to 3 and don't believe the resulting CI
- Final analysis of frequencies and CI: set ML precision to 10 and CI to 3.
- To check hillclimbing always converges on the same set of estimates: set both ML and CI levels of precision to 3

Obviously the higher the level of precision the better but there is a penalty to be paid in computing time. If the analyses are running very quickly (or you are very patient) you could set ML precision to higher levels.

3. *Running the analysis*

(i) <u>*Blood sample phenotype data i.e. input files*</u>

The data must be in a file called MHFdatafile.txt and it must be in the same directory as the program.

At present it CANNOT have more than 5000 patients (array ln_factorial would be exceeded).

The data must be in text format with columns separated by spaces
each patient is represented by a separate row of data
the following data, and no other, should be present
first column is phenotype at "codon 1"
second column is phenotupe at "codon 2"
third column is phenotype at "codon"
for these phenotypes:

- "0" indicates only wildtype is present,
- "2" mean only mutant are present
- "1" means heterozygote

4th colmun is minimum number of clones that you know are in the individual. If you do not know MOI enter the minimum i.e. '1'.

So, for example, if the patent has ony wildtype at codon 1, only mutant at codon 2 and both types (due to mixed infection) at codon 3, and it is know (e.g. from microsatellite or GLURP genotypes) that the patient contains at 4 clones, then the row for that patient will be:
0 2 1 4

And so on.

Missing data must be coded as 99 and the whole record will be ignored if missing data occurs in one of the codons being analsyed

To avoid later confusion, I strongly recommend that you keep the codons in numerical order e.g. for *dhfr*
Codon 1 is 51
Codon 2 is 59
Codon 3 is 108.

If you intend to ignore 'impossible' *dhfr* haplotypes (i.e. 51 alone, 59 alone, 51+59) then the codons must be in this sequence.

It is probably easiest to keep all the codons in an Excel worksheet, again in numerical order e.g. if you have *crt* genotyped at codons 76, 163, 220, 271 and 326 then the file would have 5 codon columns and a final column for MOI. You probably want extra columns to hold information like sample number, origin of sample, date of collection and so on. That's fine, however you want to organise it. You can then simple select

the 4 columns of required data (e.g. for particular location or dates) and cut-and – paste into the data file.

If haplotypes are defined at only one codon, the data must be in the first column. Data in the other two columsn are ignored so can be given any code prvided it is 0,1, or 2.

If haplotypes are defined at two codons, the data must be in the first and second columns. Data in the third columns are ignored so can be given any code provided it is 0,1, or 2.

(ii) *Programme output*.

Programme output should be self-expalanaory. The only conventions to understand are:

- codon genotypes are in square brackets, the first set of brackets corresponding to codon 1, second brackets are codon 2 and third brackets are codon 3.
- For codon genotypes 0=wildtype, 1=mutant
- For blood phenotypes 0=only wildtype 1= both wildtype and mutant present, 2= only mutant

So if you were examining *dhfr* haplotypes and, as suggested codon 1=position 51, codon 2=59 and codon 3=108

Haplotype [0][0][0] is wildtype
Haplotype[0][0][1] is mutant at *dhfr*108
Haplotype [1][0][1] is mutant at *dhfr*51+108
Haplotype [1][1][1] is mutant at *dhfr*51+59+108 ‘triple’ mutation.

This will menable you to interpret the estimated haplotype frequencies e.g.
Allele[0][0][0] 0.100282 CI 0.053275 to 0.165301
Allele[0][0][1] 0.036462 CI 0.010825 to 0.083509
Allele[0][1][0] 0.000004 CI 0.000000 to 0.017582
Allele[0][1][1] 0.863227 CI 0.791573 to 0.918791
Allele[1][0][0] 0.000006 CI 0.000000 to 0.017584
Allele[1][0][1] 0.000009 CI 0.000000 to 0.017587
Allele[1][1][0] 0.000005 CI 0.000000 to 0.017583
Allele[1][1][1] 0.000005 CI 0.000000 to 0.017583

So:

- *dhfr* haplotype[0][0][0] is wildtype at all codons, and its estimated freqeuncy is 10% with 95%CI from 5% to 17%.
- The ‘double’ mutant haplotype with mutations at positions 59 and 108 is encoded [0][1][1] and its estimated frequency is 86% with 95% CI of 79% to 92%.
- And so on.

Error messages generally report impossible blood sample genotypes and use the above code for the genotype e.g.

- Genotype[0][1][2][1] is a blood sample phenotype which is wildtype at codon 51, mixed wildtype and mutant at codon 59, is mutant at codon 108, and has MOI=1.It is impossible to to have a mixture of wildtype and mutants in a single clone infection (i.e. MOI=1) so an error message would be produced

- Genotype[0][0][1][1] is a blood sample which is wildtype at codon 51, wildtype at codon 59, mixed wildtypes and mutant at codon 108, and has MOI=1.

NOTE: Even when a mutation is absent from the data set its frequency will not be estimated as zero, rather it will be a very small number e.g. 0.000045 (the confidence interval should include zero). Use the first 3 decimal digits, so the appropriate estimate is actually 0.000 in this case. Note also that the upper CI limit will not be zero because some clones of that haplotype could have been present in the population but at such a low frequency that, by chance, they were not represented in the datset. [the fact that best estimate is never exactly zero arises because I have avoided any estimates of exactly zero because of worries about mathmatical conventions, divisions by zero and so on]

(iii) User-defined input parameters.

The user controls the type of analysis via a series of input parameters. These must be contained in a file called MHFparameters.txt which must be in the same directory as the program. The structure of MHFinput.txt must be exactly as shown in Figure 2.

Figure 2. The structure of MHFparameters.txt

1 ←1,2,3 for number of codons to be analysed; set to 4 if require 3 codon *dhfr* genotype omitting 'impossible' clones
9 ← level of precision required for ML estimate
3 ← level of precision required for CI estimation
8 ← maximum number of clones in any sample
n ← (must be y or n) whether 'minority' genotypes will be missed in typing
0.3 ←the detection limit if minority genotypes are missed e.g. 0.3 means genotypes present at frequency less than 30% will be missed…
n ←(must be y or n) whether MOI is known for each sample
1 ← distribution type to be used if MOI is unknown
y ← (must be y or n) whether to check hillclimbing always converges on the same ML 'peak'
n ← (must be y or n) whether to check programme accuracy by simulating datasets and checking 95% of estimates fall within the 95% CI
H <- (must be H or L in uppercase) If a dataset is simulated should it be for a High or Low transmission setting
100 ← required size of dataset for simulations to check programme accuracy
500 ← number of replicates used to check hillclimbing or programme accuracy
0 ← a redundant parameter, set to zero. [This allows later programme versions to acquire additional information without making previous input files incompatible]
0 ← a redundant parameter, set to zero.
0 ← a redundant parameter, set to zero.
0 ← a redundant parameter, set to zero
0 ← a redundant parameter, set to zero.

The following inputs must be entered in MHFparameters.txt to control the analysis.

Number of codons to be analysed. 1, 2 or 3 unsurprisingly specifies that haplotypes be defined at 1, 2 or 3 codons respectively. Note that if one codon is specified their blood phenotypes must be encoded in the first column in the input file, if 2 codons are specified, the phenotypes must be in the first two columns. Entering '4' will obtain the 3 codon haplotypes ignoring the 'impossible' *dhfr* genotypes 51 alone, 59 alone, and 51+59; if you follow this option codon 51 59 and 108 must be in input columns 1,2 and 3 respectively.

Level of precision required for ML estimate; '3' indicates 3 decimal places, '7' indicates 7 decimal and so on. This is important, not because it defines the precision of estimated frequencies but indirectly because it locates the 'peak' of maximum likelihood. This is discussed in more detail in Section 2 "Decisions required before data analysis" of these notes

Maximum number of clones in the samples. The programme runs by considering all possible permutation of genotypes in the clones in a sample. As the number of clones in the samples increase, the number of permutations grows enormously and the programme gets very slow. Set this parameter equal to the maximum number of clones observed in the dataset to minimise run times.

[I realise the programme could read max MOI from the datafile but I want to keep it as an external user-defined input to future proof the programme e.g. if MOI is unknown and the program needs to estimate the distribution of MOI.]

Whether 'minority genotypes' will be missed. (Must be entered as y or n, in lowercase). There is a worry that PCR amplification will miss alleles present at lower levels. For example if there are 5 clones in the blood, 4 of which are wildtype and 1 is mutant, then the mutant genotype may not be detected. If you think this is a problem set this parameter to 'y' and set the detection limit in the next parameter.

Detection limits for 'minority clones'. If you specify that minority genotypes will be missed (see above) this parameter gives the detection limit below which minority clones will be missed. So if it is 0.3, then alleles that constitute less than 30% of the parasites in the sample at that locus will not be detected.

Whether MOI is known for each sample. (Must be entered as y or n, in lowercase). Self-explanatory provided you know that MOI is multiplicity of infection, also known as clonal multiplicity. There is an option to allow the programme to estimate the distribution of MOI but I haven't tested it (due to a lack of interest). Contact me if you want to use this option.

Distribution type for unknown MOI. If MOI is unknown the programme will attempt to find (by maximum likelihood) a distribution of MOI that best fits the data. Its should be encoded as follows: 0=Negative Binomial, 1=Poisson, 2=conditional Poisson.

Whether to check hillclimbing always converges on the same set of estimates. (Must be entered as y or n, in lowercase). Explained in the next section i.e. "4. *Checking the analysis: check you should make"*.

Whether to check accuracy of programme. (Must be entered as y or n, in lowercase). This simulates a number of datasets (defined according to the previous parameters) to check that 95% of estimates do fall within the 95% CI. Explained in the next section i.e. "4. *Checking the analysis: check you should make"*.

If a dataset is simulated should it be for a High or Low transmission setting? For a high transmission setting the following frequencies of MOI (multiplicity of infection i.e. number of clones per sample) is:
1=10%, 2=20%, 3=30%, 4=20% 5=15% 6=5%.
And for Low transmission setting, the following frequencies of MOI are assumed:
1=60%, 2=35%, 3=5%.

Size of datasets used to check accuracy of programme. Defines the size of simulated datasets produced to check the accuracy of the program e.g. if set to 100 the datasets will each contain 100 simulated blood samples.

Number of replicates to run when checking hillclimbing or programme accuracy. Self explanatory e.g. if set to 500 will either, depending on the parameter values entered above, (i) analyse the field dataset 500 times from randomly selected initial conditions (internal programme estimates) to check they converge on the same

estimation for haplotype frequencies, or (ii) simulate 500 artificial datasets to check that 95% of estimates fall within the 95% CI.

(iv) <u>Errors and Warnings generated from the programme.</u>

A function “check_basic_calculation” runs automatically to check the computer is calculating some basic operations correctly e.g. than 0!=1, factorials are accurate etc.

A function “Check_dhfr_haplotypes” can be run to check for ‘impossible’ *dhfr* haplotypes. It is believed by many workers that mutations at 51 and 59 cannot occur in the absence of a mutation at 108. Consequently haplotypes with mutations at 51 alone, 59 alone, and 51+59 are impossible.
This function will run automatically if user has set options that (i) these haplotypes are to be ignored and (ii) that minor clones are not missed during genotyping

A function “Check_MOI” identifies records where one or more codon(s) are heterozygous but multiplicity of infection (MOI) is 1. This is obviously impossible because a codon can only be heterozygous if 2 or more clones are present in the blood sample.
This function runs automatically if user has defined that MOI is know

How the user decides to correct anomalies identified by these checks is entirely up to him/her.

There are various error checks that may flag suspicious events and cause the programme to end or to return a ridiculously low LL (e.g. -100000000). The main examples are

- To check that frequencies sum to unity and that individual frequencies do not exceed 1 or fall below 0. The programme uses floating point arithmetic so there will occasionally be rounding errors that violate these rules and the programme will terminate.
- When calculating CI, the assumed frequencies sometimes fall very close to zero and if a haplotype of that type is identified that the programme will state that this is highly unlikely and may terminate with a very low LL.

There is an obvious trade off between too many false alarms and programme robustness so I have tended to go for the latter. All the messages I have obtained so far have been false alarms e.g. “frequencies should sum to 1.0 but programme estimates sum as 0.999999”.

If the programme does terminate and the warnings seem like false alarms (e.g. rounding errors) simply run it again; it will start with different random number and it should run OK.

4. *Checking the analysis: checks you should make.*

(i) Checking convergence on frequency estimates

In the introduction I briefly noted that the method uses a 'hill climbing' routine (Figure 1B). Imagine a 3D graph where the 2 horizontal axes represent 2 haplotype frequencies. The surface plotted on the vertical axis is likelihood but this surface can be smooth with only a single peak (like Mount Fuji or Kilimanjaro), or it can be jagged with many peaks (imagine the Himalayas or something similar). If the surface is smooth and contains only a single LL 'peak' then the "Hill climbing" always moves up the hill and eventually arrive at the same maximum likelihood value irrespective of the starting estimates (i.e. the parameters from which we started the hill climb). This is the usual case but we cannot exclude the possibility that there are multiple peaks of likelihood (i.e. like the Himalayas) and that our "hill climb" just takes us up a little hill close to our starting parameters while we ignore the big 'peak' of LL somewhere else in parameter space. These false peaks are rare but I picked up one example in my model group of 117 field data sets: a 'false' peak with a lower LL was reached in about 10% of the 1000 re-analyses on this dataset and resulted in quite large differences in estimated haplotype frequency (one increasing from 0.37 to 0.5 with a corresponding fall from 0.47 to 0.33 in the other main haplotype).

Once the analysis has been obtained it is therefore important to confirm that the programme always arrives at the same 'peak' irrespective of the initial starting parameters. There is an option to confirm this by repeating the analysis from, for example, 1,000 or 10,000 randomly-generated starting conditions. I suggest you find out how long this will take (e.g. by timing a run of 100 repeats) and then work out how many you can run over the weekend, or over a week using an unemployed laptop. Obviously the larger the number of repeats, the better. Note that this is quicker than the full analyses because the programme just checks it arrives at the same 'peak' and need not calculate confidence intervals.

So, it seems good practice to just leave the programme running overnight or the weekend checking that it always converges on the same peak.

(ii) Checking programme accuracy.

I have included an option in this programme that simulates datasets so that you can check they are being analysed properly. This option:

- Looks at all the user-defined values used in the analysis (number of codons investigated, whether minor clones are missed, whether multiplicity of infection is known, etc).
- Generates random frequencies of haplotypes and then simulates a dataset of the required type and size noting exactly how many haplotypes of each type go into the simulated dataset. This ensures we know the 'true' haplotype frequencies in the simulated dataset.
- It then invokes the main programme to see how well it estimates the 'true' frequencies and whether the 'true' values fall within the 95% confidence interval.

The user can command the programme to do this numerous times (e.g. 1,000) to check the programme accuracy: it prints out ‘true’ frequency, estimated frequency with 95% CI and whether the ‘true’ frequency falls within the 95% CI. At the end of the process it prints out how often the ‘true’ frequencies fall within the 95% CI. Obviously if the programme is working well this should happen about 95% of the time. In reality 98% to 99% of true frequencies should fall within the 95% CI because the approximation used by maximum likelihood to calculate confidence intervals (i.e. a drop in 2LL units) is conservative. Remember also not to count two errors in the same simulated dataset because the frequencies must sum to one so are not independent. In the simple case of 2 alleles, if one frequency lies outside the 95%CI then so must the other one to ensure that the two frequencies sum to one.

5. Checking the analysis: checks I have made.

All computer programmes (except the most basic) contain bugs, and this one is unlikely to be an exception. As is conventional, we recognise this fact then simply cross our fingers and hope the bug(s) are not too serious. Checks I have made are as follows:

- I analysed 48 datasets to estimate mutation frequencies at single codons. I obtained the same results as Tom Smith obtaining independently using the Winbugs programme to get Bayesian estimates.

- Several of these datasets had very low frequency of mutations at other codons. When I analysed at all three codons the two principle haplotype had very similar estimated values to those obtained on the single codon estimate.

- I have simulated various datasets to check that 95% of true frequencies lie within the 95% confidence interval. I suggest you do the same when you analyse your dataset (i.e. simulate a similar sized dataset to be analysed at the required number of codons, see section 4).

Note that I have not checked the analysis when MOI is unknown. This seems fairly rare nowdays because microsatellite genotyping is routine. If you do wish to analysis a dataset where MOI is unknown I suggest you contact me.

6. How to tell if frequencies differ between datasets.

We often want to know whether frequencies observed in different surveys (e.g. from the same survey sites taken at different time points or surveys from different geographic locations) are ‘statistically significant’. The appropriate way of doing this is by a Likelihood ratio test (look it up in Wikipedia; their explanation is far clearer than anything I could manage).

To do this, first analyse the surveys separately and note the LL excluding the multinomial coefficient. Then combine the surveys into a single dataset and re-run the analysis; again note the LL excluding the multinomial coefficient. Then add the LL from the separate analyses and subtract the LL of the combined. For example if you have two surveys which separately have LL of -20 and -30 while the LL for the combined dataset is -53, then your sum will be (-20)+(-30)-(-52) = 3.

Multiplying this sum by two gives a Chi-squared statistic with degrees of freedom (df) equivalent to the difference in the number of parameters fitted.

The number of parameters fitted per analysis is the number of frequencies you estimated minus 1 (you subtract one because the ‘last’ frequency is not really estimated: its value is constrained so that total frequencies add up to one). For example is you analyse a single codon you estimate two frequencies, mutant and wildtype, so you are fitting one parameter (because if mutant frequency is 70% then wildtype must be 30%). If you are looking at all 3 codons then there are 8 haplotype frequencies so you are estimating 7 parameters.

So in the above example:

- If analysis was at a single codon then you fit 2 parameters when analysing surveys separately and a single parameter in joint analysis so the Chi squared statistic is 2x3=6 with 1 df.

- If analysis was at two codons (4 haplotypes) then you fit 6 parameters when analysing surveys separately (3 in each analysis) and three parameters in the joint analysis so the Chi squared statistic is 2x3=6 with 3 df.

Similarly, if you analyse 3 surveys for the dhfr haplotypes (i.e. excluding the 3 impossible haplotypes) you are estimating 5 frequencies so using 4 parameters. The separate analyses use 3x4=12 parameters while the single combined analysis uses 4 so the statistic would have 8df. Assume the LL for the separate analyses were -10, -20, -30 and for the combined was -72 then the Chi squared would be 2x12=24 with 8df

And so on.

You need to look up a table of Chi-squared to test for significance.

	**P**					
**DF**	**0.05**	**0.02**	**0.01**	**0.005**	**0.002**	**0.001**
**1**	3.841	5.412	6.635	7.879	9.549	10.828
**2**	5.991	7.824	9.210	10.597	12.429	13.815
**3**	7.815	9.837	11.345	12.838	14.795	16.266

**4**	9.488	11.668	13.277	14.860	16.924	18.467
**5**	11.070	13.388	15.086	16.750	18.907	20.515
**6**	12.592	15.033	16.812	18.548	20.791	22.458
**7**	14.067	16.622	18.475	20.278	22.601	24.322
**8**	15.507	18.168	20.090	21.955	24.352	26.124
**9**	16.919	19.679	21.666	23.589	26.056	27.877
**10**	18.307	21.161	23.209	25.188	27.722	29.588
**11**	19.675	22.618	24.725	26.757	29.354	31.264
**12**	21.026	24.054	26.217	28.299	30.957	32.909
**13**	22.362	25.472	27.688	29.819	32.535	34.528
**14**	23.685	26.873	29.141	31.319	34.091	36.123
**15**	24.996	28.260	30.578	32.801	35.628	37.697
**16**	26.296	29.633	32.000	34.267	37.146	39.252
**17**	27.587	30.995	33.409	35.718	38.648	40.790
**18**	28.869	32.346	34.805	37.156	40.136	42.312
**19**	30.143	33.687	36.191	38.582	41.610	43.820
**20**	31.410	35.020	37.566	39.997	43.072	45.315

So
The analysis with Chi-squared of 6 and 1df is statistically significant with $p<0.02$
The analysis with Chi-squared of 6 and 3df is not statistically significant
The analysis with Chi-squared of 24 and 8df is statistically significant with $p<0.005$
And so on.

If you get onto the web you can usually find a page that will give you exact 'p' values if you enter the Chi-squered value and d.f.

7. Acknowledgements.

Bill Watkins persuading me to start writing this programme around 1998 and I cursed him strong and long as the amount of code and user-defined options escalated. Tom Smith persuaded me to resurrect it in 2007 and to develop it as a general tool for analysis. I am grateful to them both.

Appendix 1: The MalHaploFreq algorithm.

The basic algebraic description for the single locus case is as follows.

The estimate of mutant allele frequency is f. There are three possible phenotypes, θ [all wildtype (θ=0), mixed wildtype+mutant (θ=1), all mutant (θ=2)] and numerous values of MOI, μ, and each phenotype has an equation of type

$$p(\theta,\mu) = \sum_{c=1}^{m}\sum_{j=0}^{c}\left(\frac{c}{j(c-j)}\right) f^{j}(1-f)^{(c-j)}w \qquad \text{(A1.1)}$$

Where:
$p(\theta,\mu)$ is the probability of observing phenotype θ for any given value of μ.
c is MOI
m is the maximum MOI observed in the dataset
j is the number of mutant clones in the sample (so that *c-j* is the number of wildtype clones)
w is an indicator which takes the value 1 if that combination of clones gives rise to phenotype θ and c=μ else it takes value 0.

Note that
(1) The summation over c is redundant because we could simply set c=μ. I have kept the redundancy because this is how the computer programme works ie. cycling all values of c and all possible haplotype combinations within *c* and then using the indicator variable *w* to assign that haplotype combination probability to the appropriate phenotype probability p(θ,μ).
(2) We are not yet working in logs.
(3) The programme checks that $p(0,\mu)+p(1,\mu)+p(2,\mu)=1$ i.e. that the probabilities of the 3 phenotypes sums to unity within each value of MOI, μ.

We now have the expected probability of each phenotype $p(\theta,\mu)$ and the number of each phenotype $n(\theta,\mu)$ observed in the dataset. The log likelihood, LL, of obtaining the observed dataset is obtained as

$$LL = \sum_{\mu=1}^{m}\ln(t_\mu!) + \sum_{\theta=0}^{2} - \ln[n(\theta,\mu)!] + \ln\,[p(\theta,u)]n(\theta,\mu) \qquad \text{(A1.2)}$$

Where t_μ is the total number of observations in the dataset with MOI=μ. Note that the probabilities of phenotypes sum to unity within each MOI class so the multinomial coefficient has to be calculated for each MOI.

The summation just serves to incorporate all the possible phenotypes into the multinomial probability of observing the dataset. This is best illustrated by considering the simple case where MOI can be only 1 or 2, giving 5 phenotypes in total (because it is impossible to have a mixed mutant+wildtype infection if MOI=1). The probability of observing a given dataset is

$$(\frac{t_1!}{n(0,1)!\,n(2,1)!})p(0,1)^{n(0,1)}p(2,1)^{n(2,1)}(\frac{t_2!}{n(0,2)!\,n(1,2)!\,n(2,2)!})p(0,2)^{n(0,2)}p(1,2)^{n(1,2)}p(2,2)^{n(2,2)}$$

(Equation A1.3)

Equation A1.2 also considers haplotypes which are not observed in the dataset. They make no difference to the LL because n(θ,μ)=0 means the second term in the summation is zero and the conventions that 0!=1 and ln(1)=0 mean that the first term in the summation is also zero.

(A) How the programme calculates LL for any set of guestimates for the parameters

(1) Step 1: calculate the probability of observing each blood sample phenotype

The programme will consider each number of clones (MOI; multiplicity of infection) in turn and cycles through all the combinations of the eight haplotypes that can occur within that MOI. For example, if there are 5 clones, one possible combination would be 3 haplotypes of type [1][1][1] i.e. mutant at all codons
1 haplotypes of type [0][1][1] i.e. wildtype at codon 1, mutant at codons 2 and 3
1 haplotype of type [1][1][0] etc

This would give a phenotypes of [1][2][1][5] i.e.wildtype and mutants present at codon 1, only mutant at codon 2, wt and mutants present at codon 3, with MOI of 5.

The probability of getting this combination of haplotypes is obtained from the multinomial distribution i.e.

$\binom{5}{3,1,1} 3^x 1^y 1^z$ where x,y,z are the current guestimates for the frequencies of haplotypes [1][1][1], [0][1][1] and [1][1][0] respectively and the multinomial coefficient

$$\binom{5}{3,1,1} = \frac{5!}{3!1!1!}$$

A running total of probabilities of observing the various possible phenotypes combinations is kept stored in an array prob[][][][] where the final index is MOI.

So in the above example
Prob[1][2][1][5] would be incremented by the solution to Equation 1.
The reason they are incremented is that different combinations of haplotypes can give rise to the same observed phenotype.

If minority clones are missed, this is where the effect is incorporated. In the above example, assume clones present at freq less than 0.33 are missed then the phenotype [1][2][1][5] would become [2][2][2][5] because the single clones with wildtype at codons 1 and 3 are below the detection limit.

If MOI is unknown. The programme will go through the above process then move all the probabilities back into the array prob indexed as MOI=1. This is achieved by weighting the original calculation by the probability of getting that MOI given the current estimates of mean MOI (and dispersal coefficient if considering the negative binomial). In the above example

If Prob[1][0][1][5]=0.1 and probability of having MOI=5 is 0.2 then
Prob[1][0][1][1] is incremented by 01x0.2

At one stage I was considering also using index MOI=2 when MOI is unkown because this is the minimum number of clones that are present if one of the codon genotypes is heterozygous. On reflection I don't think this is necessary because as mean MOI increases so will the number of phenotypes with heterozygous codons so I think the calculations already make use of this information.

What I have not managed to incorporate is the situation when all the codons being considered in the current analysis are homozygous, so it may be that only a single clone is present, but it is known that at least two clones are present because a phenotype not included in the analysis is known to be heterozygous. This is very tricky because the information provided by the second locus would depend on the allele frequencies and MOI of infection which both affect the probability of obesrving a heterozygote

(2) Step 2: calculate the probability of observing the given dataset

Assume there are:
20 patients with MOI=1 among which 15 are [0][0][0], 3 are [0][0][2] and 2 are [2][0][2]
10 patients with MOI=2 among which 5 are [0][0][0], 4 are [0][0][1] and 1 is [1][0][1]
Etc

Then the probability, or likelihood, of observing the dataset is

$$\binom{20}{15,3,2} prob[0][0][0][1]^{15}\, prob[0][0][2][1]^{3}\, prob[2][0][2][1]^{2}$$

$$\times \binom{10}{5,4,1} prob[0][0][0][2]^{5}\, prob[0][0][1][2]^{4}\, prob[1][0][1][2]^{1}$$

x etc.
And so on for each MOI.

If the MOI is unknown then all genotypes are stored assuming MOI=1 so only the first term in the above equation is required

Important: Note that the multinomial coefficient will also contain zeros because each possible phenotype is investigated and some will have zero observations. In effect

$\binom{20}{15,3,2}$ becomes $\binom{20}{15,3,2,0,0,0....}$ so it is vital that 0!=1 (and that ln(1)=0) to keep the calculations correct.

Also some of the power terms may be zero eg $prob[0][0][1][3]^0$ so it is vital that any number, including zero, raised to the power zero obeys the convention that it equals unity.

Appendix 2. Alternative algorithm.

This alternative algorithm was suggested by Prof. Tom Smith and Dr. Melissa Penny at the Swiss Tropical Institute, Basle. It has the advantage of being algebraically more transparent.

[on a technical note they use a different coding for blood phenotypes i.e. 1 is all wildtype, 2 is all mutant, 3 is mixed. Here we stick to the coding used in MalHaploFreq i.e.
1= all wildtype
2=mixed
3 is all mutant]

<u>*(i) One locus case.*</u>
As in Hill & Babiker (3) and Schneider *et al* (4).

	wildtype	mixed	mutant
Probability	$P(1) = (1\text{-}f)^n$	$P(2)=1\text{-}P(1)\text{-}P(3)$	$P(3)=f^n$

Where
f= frequency of the mutation
n is multiplicity of infection.

Each of the N samples are then examined in turn using indicator variables:
W_{1i} taking the value 1 where the wild type allele is present, and 0 where it is not.
M_{1i} taking the value 1 where the mutant allele is present, and 0 where it is not.

The likelihood associated with the vector of allele frequencies, (x_1, x_2) is calculated using the indicator variables to determine which of the terms is included in the product for each sample, i, i.e.:

$$L(x_1 x_1) = \prod_{i=1}^{N} W_{1i}\,(1 - M_{1i})P(1) + M_{1i}W_{1i}(p2) + M_{1i}(1 - W_{1i})P(3)$$

Or equivalently, the log likelihood:

$$Log\ L(x_1 x_1) = \sum_{i=1}^{N} \log\,[W_{1i}(1 - M_{1i})P(1) + M_{1i}W_{1i}(p2) + M_{1i}(1 - W_{1i})P(3)]$$

This illustrates the differences between the two algorithms. This Smith and Penny approach examines (using an indicator variable) and incorporates each of the N observed phenotypes sequentially. The algorithm used in MalHaploFreq is fundamentally different. It systematically investigates all possible combinations of haplotypes and works out what phenotype would arise from that combination. It keeps a running total of the frequency of each phenotype that occurs under the current estimates of haplotype frequencies. When all possible combinations have been

examined the running total gives the probability of each phenotype. It then uses the multinomial distribution to find the likelihood of observing the phenotypes that occur in the dataset.

The MalHaploFreq approach is more cumbersome because it will work out the probability of many phenotypes that will not be observed in the dataset but it has two distinct advantages

- *Flexibility. The haplotypes may not give rise to the predicted phenotype if 'minority' clones are not detected in genotyping. For example if a person has 5 clones of which 4 are wildtype and 1 mutant, then the single mutant clones may be swamped during genotyping so that the phenotype is scored as 'pure wildtype'. Similarly, if MOI is unknown it is simple to process and merge the different MOI classes that may give rise to the same phenotype.*
- *Robustness. When 3-locus haplotypes are considered then there are 27 distinct blood phenotypes, each of which requires a separate equation under the Smith and Penny algorithm. These may become complex (see later) and encoding them runs the risk (at least for me) of introducing errors.*

Obviously the two algorithms should give identical results.

(ii) The 2 locus case.

This is conceptually similar to the one locus case. The haplotype frequencies are represented as follows:

		Genotype at second locus	
		Wild type	Mutant
Genotype at first locus	Wild type	x_{11}	x_{12}
	Mutant	x_{21}	x_{22}

Giving rise to the following phenotype probabilities:

		Phenotype at second locus		
		Wild type	Mixed	Mutant
Phenotype at first locus	Wild type	$P(1,1) = x_{11}^n$	$P(1,2) = (x_{11} + x_{12})^n - x_{11}^n - x_{12}^n$	$P(1,3) = x_{12}^n$
	Mixed	$P(2,1) = (x_{11} + x_{21})^n - x_{11}^n - x_{21}^n$	$P(2,2) = 1 - (x_{11} + x_{21})^n - (x_{11} + x_{12})^n - (x_{21} + x_{22})^n - (x_{12} + x_{22})^n$	$P(2,3) = (x_{12} + x_{22})^n - x_{12}^n - x_{22}^n$
	Mutant	$P(3,1) = x_{21}^n$	$P(3,2) = (x_{21} + x_{22})^n - x_{21}^n - x_{22}^n$	$P(3,3) = x_{22}^n$

Note the algorithm for mixed infections. The sum of frequencies that can give rise to mixed infection are raised to the power of MOI. This gives the total probabilities of all the combinations of these haplotypes so it is necessary to then subtract those

phenotypes that are not mixed. For example P(2,1) is mixed at codon 1 and wildtype at codon 2. These can only arise from combinations of haplotypes x_{11} and x_{21} and $(x_{11}+x_{21})^n$ gives the frequency of all their possible combinations. The frequency of the non-mixed phenotypes is then subtracted i.e. x_{11}^n (all wildtype at codon 1) and x_{21}^n (all mutant at codon 1) giving

$$P(2,1) = (x_{11} + x_{21})^n - x_{11}^n - x_{21}^n$$

(iii) The 3 codon case.

The same notation for haplotypes is used as before i.e. an 'x' with subscripts denoting genotype at the three codons so that, for example, x_{212} is mutant at codons 1 and 3 and wildtype at codon 2.

As before, the phenotype probabilities are straightforward for samples without mixed infections, e.g. the probability of observing only mutant at the first and third loci, and wild type at the second is: $P(3,1,3) = x_{212}^n$

When there are mixed infections, the same approach was used as in the two locus case i.e.

- The haplotypes that could contribute to the observed phenotype are identified.
- They are raised to the power of the MOI to get the total frequency of all possible combination of these haplotypes
- The frequencies of 'simpler' phenotypes arising from this set of haplotypes are then removed (simpler is here defined as containing mixed infections at fewer loci than the phenotype in question).

So, for example,

$$P(3,1,2) = (x_{211} + x_{212})^n - x_{211}^n - x_{212}^n = (x_{211} + x_{212})^n - P(3,1,1) - p(3,1,3)$$

and

$$P(3,2,2) = (x_{211} + x_{212} + x_{221} + x_{222})^n - P(3,1,1) - p(3,1,3) - p(3,1,2) - p(3,3,1) - p(3,3,3) - p(3,3,2)$$

Setting up indicator variables *I* which take the following values

$I_i\ \ j_1, j_2, j_3\ \ = 1$ if sample *i* has phenotype $\ j_1, j_2, j_3$

$I_i\ \ j_1, j_2, j_3\ \ = 0$ otherwise

allows the log likelihood to be calculated as:

$$\log L\ \ \underline{x}\ \ = \sum_{i=1}^{N} \sum_{j_1=1}^{3} \sum_{j_2=1}^{3} \sum_{j_3=1}^{3} I_i\ \ j_1, j_2, j_3\ \ \log P_i\ \ j_1, j_2, j_3$$

where $\underline{x}$ is the vector of three locus haplotype frequencies.

Appendix 3. The original version of the Smith and Penny algorithm.

[In case I misrepresented it: This is a verbatim record of what TS Emailed to IH. <u>*Remember their coding is different*</u> as explained in Appendix 2.]

Consider initially one locus, and some set of *N* samples, indexed with *i*=1,2,..*N*. Let n_i be the MOI of sample *i*, There are then three possible phenotypes, the probabilities of which can be expressed in terms of the allele frequencies of the wild type x_1 and of the mutant $x_2 = 1 - x_1$ as follows (Table 1):

Table 1. Phenotypes, one locus case

	Wild type	Mutant	Mixture
Probability	$P(1) = x_1^{n_i}$	$P(2) = x_2^{n_i}$	$P(3) = 1 - x_1^{n_i} - x_2^{n_i}$

Let W_{1i} be an indicator variable taking the value 1 where the wild type allele is present, and 0 where it is not. Correspondingly, let M_{1i} be an indicator variable taking the value 1 where the mutant allele is present, and 0 where it is not. The vector of allele frequencies, (x_1, x_2) is then estimated by maximizing the likelihood, where the weights are used to determine which of the terms is included in the product for each sample, *i*, i.e.:

$$L\;\; x_1, x_2 \;\; = \prod_{i=1}^{N} \;\; W_{1i}\;\; 1 - M_{1i}\;\; P(1) + M_{1i}\;\; 1 - W_{1i}\;\; P(2) + M_{1i} W_{1i} P(3)$$

Or equivalently, the log likelihood:

$$\log L\;\; x_1, x_2 \;\; = \sum_{i=1}^{N} \log\;\; W_{1i}\;\; 1 - M_{1i}\;\; P(1) + M_{1i}\;\; 1 - W_{1i}\;\; P(2) + M_{1i} W_{1i} P(3)$$

Consider now the two locus situation. There are now four possible haplotypes, with frequencies as given in Table 2.

Table 2. Haplotype frequencies (2 locus case)

		Genotype at second locus	
		Wild type	Mutant
Genotype at first locus	Wild type	x_{11}	x_{12}
	Mutant	x_{21}	x_{22}

and the two locus linkage disequilibrium is then measured by :

$$D = x_{11} - \;\; x_{21} + x_{22} \;\;\; x_{12} + x_{22} \;\; ,$$

This, together with allele frequencies and the constraint $x_{11} + x_{21} + x_{12} + x_{22} = 1$, uniquely determines the four haplotype frequencies. Corresponding to these four haplotypes, 9 distinct phenotypes can be observed[1] with the probabilities given in Table 3:

Table 3. Phenotypes, two locus case

		Phenotype at second locus		
		Wild type	Mutant	Mixed

[1] For any n_i greater than 1. For n_i equal to 1 the 5 phenotypes with mixed infections should not occur.

Phenotype at first locus	Wild type	$P_i(1,1) = x_{11}^{n_i}$	$P_i(1,2) = x_{12}^{n_i}$	$P_i(1,3) = \left(x_{11} + x_{12}\right)^{n_i} - x_{11}^{n_i} - x_{12}^{n_i}$
	Mutant	$P_i(2,1) = x_{21}^{n_i}$	$P_i(2,2) = x_{22}^{n_i}$	$P_i(2,3) = \left(x_{21} + x_{22}\right)^{n_i} - x_{21}^{n_i} - x_{22}^{n_i}$
	Mixed	$P_i(3,1) = \left(x_{11} + x_{21}\right)^{n_i} - x_{11}^{n_i} - x_{21}^{n_i}$	$P_i(3,2) = \left(x_{12} + x_{22}\right)^{n_i} - x_{12}^{n_i} - x_{22}^{n_i}$	$P_i(3,3) = 1 - \left(x_{11} + x_{21}\right)^{n_i} - \left(x_{11} + x_{12}\right)^{n_i} - \left(x_{21} + x_{22}\right)^{n_i} - \left(x_{12} + x_{22}\right)^{n_i}$

In the three locus case, there are 8 possible haplotypes corresponding to … disequilibrium parameters. The corresponding formulae for the phenotype probabilities are straightforward for samples without mixed infections, where all co-infections have the same haplotype, thus for example, the probability of observing only mutant at the first and third loci, and wild type at the second is:

$$P_i(2,1,2) = x_{212}^{n_i}$$

When there are mixed infections, then the phenotype probability can be resolved into two components, (i) the sum of the haplotype frequencies of those candidate haplotypes that might contribute to the observed phenotype, raised to the power of the MOI. (ii) the sum of the probabilities for all simpler phenotypes that are compatible with this set of haplotypes (simpler is here defined as containing mixed infections at fewer loci than the phenotype in question). The second component is then subtracted from the first component. E.g.:

$$P_i(2,1,3) = \left(x_{211} + x_{212}\right)^{n_i} - x_{211}^{n_i} - x_{212}^{n_i}$$
$$= \left(x_{211} + x_{212}\right)^{n_i} - P_i(2,1,1) - P_i(2,1,2)$$

or:

$$P_i(2,3,3) = \left(x_{211} + x_{212} + x_{221} + x_{222}\right)^{n_i} - P_i(2,1,1) - P_i(2,1,2)$$
$$- P_i(2,1,3) - P_i(2,2,1) - P_i(2,2,2) - P_i(2,2,3)$$

The log likelihood is then calculated as:

$$\log L\left(\underline{x}\right) = \sum_{i=1}^{N} \sum_{j_1=1}^{3} \sum_{j_2=1}^{3} \sum_{j_3=1}^{3} I_i\left(j_1, j_2, j_3\right) \log P_i\left(j_1, j_2, j_3\right)$$

where $\underline{x}$ is the vector of three locus haplotype frequencies, and the indicator variable, $I_i\left(j_1, j_2, j_3\right)$ takes the value 1 if sample *i* has phenotype $\left(j_1, j_2, j_3\right)$ and zero otherwise.

Appendix 4. Simple tests to check programme is working

Try the simplest case, analysing the following dataset:

0 0 0 1
0 0 0 1
2 0 0 1
2 0 0 1
2 0 0 1

i.e. 5 samples each with single clone infections of which two are wildtype and three are mutant. Obviously the frequency of the wildtype is 0.4 and the mutant is 0.6

The probability of getting this is

$$\left(\frac{5!}{3!\,2!}\right)0.4^2 0.6^3 = 0.3456$$

The binomial coefficient has value 120/(6x2)= 10

0.4^2x0.6^3=0.03456

So overall max LL is ln(0.3456) = -1.0624
Value of the coefficient is ln(10)= 2.302
LL excluding coefficient is ln(0.03456)= -3.36

Appendix 5. History of programme versions and updates

Version 1.1.1

- Random number generator is now seeded from time rather than a constant. This allows users to check for convergence using different machines or processors or runs. For example, to check convergence from 1000 simulations could run 500 on different machines (this obviously requires the runs being seeded with different random numbers). The programme was seeded differently up to version 1.1.0 , i.e. during development, and seeding was a 'quick fix' used to get the programme to run on any machine
- There was a shortcut in the hillclimb routine that jumped to the next precision level when LL was close to maximum (within one unit) this shortcut was removed to make the analyse more robust in small sample sizes where a change of 1 in LL could be relatively large. A bug was identified and fixed: once the programme had broken out of one precision level it broke out of all the subsequent ones (the counter was not reset to zero)
- The best log likelihood is now presented with and without the multinomial coefficient. This should allow likelihood ratios to be calculated to check whether frequencies differ between samples as described in the main text
- There was a small bug fix. Previous to this version blank lines at the end of the input file would be regarded as repeat records of the last line (the programme did not detect end of file in these lines so continued to use the last parameter values for each line). The current version has a routine to check length of lines and should pick up these problems and print a warning.
- The routine to simulate datasets to check MalHaploFreq estimates fall within 95% CI has been updated so it can simulate datasets where minor clones are missed during genotyping.

Reference List

1. Hartl, D. L. & Clark, A. G. (1997) *Principles of Population Genetics* (Sinauer Associates, Sunderland, Ma, USA).

2. Williams, B. G. & Dye, C. (1994) *Parasitol. Today* **10**, 489-493.

3. Hill, W. G. & Babiker, H. A. (1995) *Proc. R. Soc. London Ser. B.* **262**, 249-257.

4. Schneider, A. G., Premji, Z., Felger, I., Smith, T., Abdulla, S. & Beck, H.-P. M. H. (2002) *Infect. Genet. Evol.* **1**, 183-189.
